# Supplementary material for: Decrease in Mucosal IL17A, IFNγ and IL10 Expressions in Active Crohn’s Disease Patients Treated with High-Dose Vitamin D Alone or Combined with Infliximab
Source: Nutrients. 2020 Nov 30;12(12):3699. doi: 10.3390/nu12123699 (PMC7759913; doi:10.3390/nu12123699)
Supplement: Supplementary file 1 [file nutrients-12-03699-s001.zip › Supplementary materials 05.11.20.docx]

**Supplementary materials**

Table S1. Other exclusion criteria

| **Parameter** |
| --- |
| Inability to understand Danish |
| Allergy to Dekristol |
| Allergy to Infliximab |
| Allergy to soy |
| Allergy to peanuts |
| Benzothithiadiazine derivate treatment |
| Phenytoin treatment |
| Barbiturate treatment |
| Digoxin treatment |
| Sarcoidosis |
| Moderate to severe heart failure |
| Demyelinating diseases including Guillian-Barré and Multiple Sclerosis |
| Immobilisation |
| Hypernatremia |
| Hyperchloremia |
| Cancer |
| Vaccinated with a live vaccine within four weeks of screening |

**Table S1.** Additional exclusion criteria for included patients.

Table S2. Taqman probes

| Gene | Clone |
| --- | --- |
| B2M | Hs00187842_m1 |
| VDR | Hs00172113_m1 |
| RPLPO | HHs99999902_m1 |
| IL-17A | Hs00174383_m1 |
| IL-17F | Hs00369400_m1 |
| CAMP | Hs00189038_m1 |
| IL-22 | Hs01574154_m1 |
| IL-10 | Hs00961622_m1 |
| IFN-gamma | Hs00989291_m1 |
| TNF-alpha | Hs01113624_g1 |
| TGF-beta | Hs00998133_m1 |
| RPS9 | Hs02339424_g1 |

Table S3. Adverse events.

| **Adverse event (AE)** | **Ifx+VitD**  **(N = 8)** | **Ifx+placebo-VitD**  **(N = 8)** | **Placebo-Ifx+VitD**  **(N = 16)** | **Placebo-Ifx+placebo-VitD**  **(N = 8)** |
| --- | --- | --- | --- | --- |
| Nausea | 1(3) |  | 1 |  |
| Gastroenteritis |  |  | 2 |  |
| Abdominal pain |  | 1 | 3 | 1 |
| Threadworm |  |  | 1 |  |
| Pharyngitis |  | 3 | 1 |  |
| Dermatitis |  |  | 1 |  |
| Pruritus |  |  | 1 |  |
| Conjunctivitis |  | 1 | 1 |  |
| Fatigue | 1 |  |  |  |
| Iron deficiency |  | 1 |  |  |
| Bacterial overgrowth |  | 1 |  |  |
| Dizziness |  |  | 1 |  |
| Flatulence |  |  | 1 |  |
| Hidrosadenitis |  |  | 1 |  |
| Influenza | 1 |  |  |  |
| Hypophosphatemia | 1 |  |  |  |
| Vomiting |  | 1 |  |  |
| Coughing |  |  |  | 1 |
| Insomnia |  |  |  | 1 |
| Cystitis |  |  |  | 1 |
| Acne |  |  |  | 1 |
| **Total AEs** | **4** | **8** | **14** | **5** |
| **Patients with 1 or more AE, n** | **4** | **5** | **7** | **4** |
| **Serious adverse events** | | | | |
| Allergic infusion reaction | 1 |  |  |  |
| Observation for allergic reaction, discomfirmed | 1 |  |  |  |
| Observation for perforation, discomfirmed |  |  |  | 1 |
| Fibrostenosis operation |  | 1 |  |  |
| **Total SAE** | **2** | **1** |  | **1** |

**Table S3.** Adverse events and serious adverse events during weeks 0 to 7. Adverse events are listed according to MedDRA version 10 dictionary.

SAE - serious adverse event; AE - adverse event; n - number.
